# Valorization of Turnip Greens (*Brassica rapa* subsp. *sylvestris*) Wastes: Investigation on the Sustainable Recovery of Bioactive Extracts with Antioxidant and Antibiofilm Properties

**DOI:** 10.3390/molecules31020388

**Published:** 2026-01-22

**Authors:** Anna Maria Maurelli, Davide Coniglio, Francesco Milano, Sara Mancarella, Barbara Laddomada, Vincenzo De Leo, Francesco Longobardi, Francesca Coppola, Florinda Fratianni, Michelangelo Pascale, Filomena Nazzaro, Lucia Catucci

**Affiliations:** 1 Institute of Food Sciences, Italian National Research Council (CNR-ISA), URT-Bari, Via Orabona 4, 70126 Bari, Italy; annamaria.maurelli@isa.cnr.it; 2 Department of Chemistry, University of Bari Aldo Moro, Via Orabona 4, 70126 Bari, Italy; davide.coniglio@uniba.it (D.C.); francesco.longobardi@uniba.it (F.L.); lucia.catucci@uniba.it (L.C.); 3 Institute of Sciences of Food Production, Italian National Research Council (CNR-ISPA), S.P. Lecce-Monteroni, 73100 Lecce, Italy; francesco.milano@cnr.it (F.M.); saramancarella@cnr.it (S.M.); 4 Institute of Sciences of Food Production, Italian National Research Council (CNR-ISPA), Via Amendola, 122/O, 70126 Bari, Italy; barbara.laddomada@cnr.it; 5 Department of Agricultural Sciences, University of Naples Federico II, Piazza Carlo di Borbone 1, 80055 Portici, Italy; francesca.coppola2@unina.it; 6 Institute of Food Sciences, Italian National Research Council (CNR-ISA), Via Roma 64, 83100 Avellino, Italy; florinda.fratianni@isa.cnr.it (F.F.); michelangelo.pascale@cnr.it (M.P.); filomena.nazzaro@cnr.it (F.N.)

**Keywords:** *Brassica rapa* subsp. *sylvestris*, agricultural waste, Design of Experiments, antioxidant extraction, ultrasound-assisted extraction, polyphenols, phenolic acids, antioxidant activity, antimicrobial activity, biofilm inhibition

## Abstract

The valorization of agri-food residues is crucial for advancing circular bioeconomy strategies and mitigating environmental impacts. Turnip greens (*Brassica rapa* subsp. *sylvestris*) are a traditional vegetable cultivated in southern Italy. While the edible portions include flower sprouts, buds, and young leaves, the more leathery leaves and stems are typically discarded. These wastes represent valuable sources of compounds with antioxidant and antimicrobial potential. This study aims to develop the extraction of phenolic compounds from turnip green residues using two techniques: silent maceration and ultrasound-assisted extraction (UAE). Ethanol was selected over methanol as a food-safe alternative solvent, with preliminary tests confirming equivalent efficiency. A Design of Experiments (DoE) approach was applied to both leaves and stems to assess the effects of solvent composition, solvent-to-matrix ratio, and extraction time on Total Phenolic Content and Trolox Equivalent Antioxidant Capacity. DoE results identified UAE as the most effective method for stems, while for leaves, the solvent-to-dry-mass ratio was the key parameter. HPLC-DAD analysis was performed to identify and quantify the phenolic acids in selected extracts. The antibacterial activity of these extracts against biofilms of six pathogenic strains was evaluated using crystal violet and MTT assays, confirming efficacy in both biofilm formation and mature stages.

## 1. Introduction

The agri-food industry has a significant environmental footprint, often even broader and more complex than agricultural production alone. This is because it includes all stages from harvesting to processing, including packaging, distribution, and waste disposal. The main environmental impacts arise from energy consumption (required for harvesting, processing, refrigeration, and transportation of agricultural products), intensive water use, and waste production.

The valorization of agri-food waste biomass represents a valid strategy for minimizing environmental impacts and a necessary action for the transition from a linear to a circular economy model, as well as an opportunity for economic development and employment. Well-established options for recovering biomass waste include the production of renewable energy (through combustion and biogas production) [1], animal feed [2], fertilizers and soil improvers (through composting) [3], and biobased materials (such as bioplastics or insulating panels for buildings) [4]. Emerging perspectives include the valorization of waste biomass to obtain valuable chemical intermediates, solvents [5], syngas, biofuels [6], and even composite materials and nanomaterials [7]. Furthermore, waste from agricultural products represents a source of high-value and nutritionally important biomolecules. For example, leaves, peels, stems, seeds, etc., have been used to extract bioactive compounds such as polyphenols [8], flavonoids [9], carotenoids [10], fibers [11], essential oils [12], and other secondary metabolites [13] that can have important applications in various sectors, from food to pharmaceuticals and cosmetics.

Crops belonging to the Brassicaceae (or cruciferous) family constitute one of the most economically important horticultural groups globally. According to data provided by the Food and Agriculture Organization of the United Nations (FAO), global production of the main edible Brassicaceae, including cabbage, cauliflower, and broccoli, reached approximately 100.3 million tons in 2023, on a total cultivated area of approximately 4 million ha [14]. Epidemiological studies have shown that consumption of cruciferous vegetables is associated with a reduced risk of chronic diseases, particularly age-related diseases, cancer, Alzheimer’s disease, and cataracts [15]. However, cruciferous waste and byproducts (*viz.*, leaves, stalks, and stems) range between 30 and 50% [16], representing a significant economic and environmental challenge. These byproducts represent a potential source of low-cost and readily available bioactive compounds that can be recovered and exploited through appropriate valorization processes [17]. Although the phytochemical profile depends on the specific cultivar of the crop and on climatic and agronomic conditions, bioactive secondary metabolites such as glucosinolates and phenolic constituents, including flavonoids, anthocyanins, carotenoids, and tocopherols, can be recovered from cruciferous vegetables [17,18,19,20]. Among these bioactive compounds, phenolic constituents—including phenolic acids, flavonoids, and hydroxycinnamic acid derivatives—represent a major fraction and are of particular interest due to their well-documented antioxidant, anti-inflammatory, and antimicrobial properties [21]. Traditional extraction methods for Brassicaceae typically employ aqueous alcoholic solvents (mainly methanol-water) [22]. However, substituting methanol with ethanol—classified as Generally Recognized as Safe (GRAS)—aligns extraction processes with green chemistry principles and enables the production of food-grade bioactive extracts suitable for functional food and nutraceutical applications.

Moreover, modern extraction optimization requires rational exploration of the multidimensional parameter space to identify synergistic effects and optimal conditions specific to each plant matrix. Design of Experiments (DoE) methodologies enable such systematic optimization by simultaneously evaluating multiple factors and their interactions, thereby achieving fine control over extraction efficiency while minimizing experimental effort.

In this work, wastes from turnip greens (*Brassica rapa* subsp. *sylvestris*, Broccolo group) were valorized to obtain bioactive compounds. Turnip greens are a cruciferous vegetable grown primarily in southern Italy (approximately 10,000 ha of cultivated land, yielding about 150,000 tons in 2024 [23]), where they are known as *Cime di rapa*. In addition to the domestic market, part of the production is exported mainly to Europe, Canada, and the United States, where this vegetable is also known as broccoli rabe, broccoli de rabe, rapini, etc. [24]. Considering the favorable balance between its performance and the simplicity of the equipment, ultrasound-assisted extraction (UAE) was chosen over other novel extraction aids and compared with silent maceration (SM) on different fractions of turnip greens waste (the tougher, leathery leaves and stems) to enhance the phenolic content and antioxidant properties of the extracts [25]. The DoE approach was used to evaluate the impact of solvent composition, solvent/matrix ratio, and extraction time on the Total Phenolic Content (TPC) and Trolox Equivalent Antioxidant Capacity (TEAC) of the extracts obtained from leaves and stems using SM and UAE. Selected extracts obtained from leaves and stems were then characterized for phenolic acid content and tested for antimicrobial and antibiofilm activity.

## 2. Results and Discussion

### 2.1. Preparation and Characterization of the Plant Waste Material

**Figure 1:**
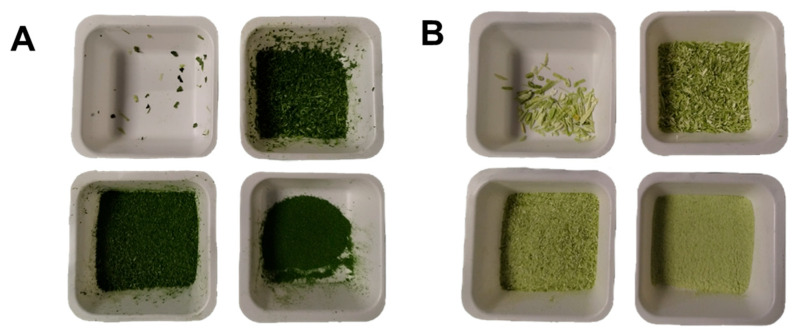
Photographs of (**A**) leaves and (**B**) stems, shredded and sorted by particle size after using calibrated sieves.

After being separated and freeze-dried, the waste leaves and stems of turnip greens were crushed and reduced into a powder (Figure 1). A preliminary analysis of the particle size was conducted as described in Section 3.2. For both sample types, the most abundant fraction was that with a particle size between 0.5 and 1 mm (Table 1). The stems produced a more consistent fraction of fragments with a diameter greater than 2 mm (Table 1 and Figure 1B), due to the more abundant presence of tougher and insoluble fibers [26], especially in the external part. The powders thus obtained were used for extraction tests conducted using the classical SM and UAE according to the DoE described in the next paragraph.

### 2.2. Parameters Screening by DoE

**Figure 2:**
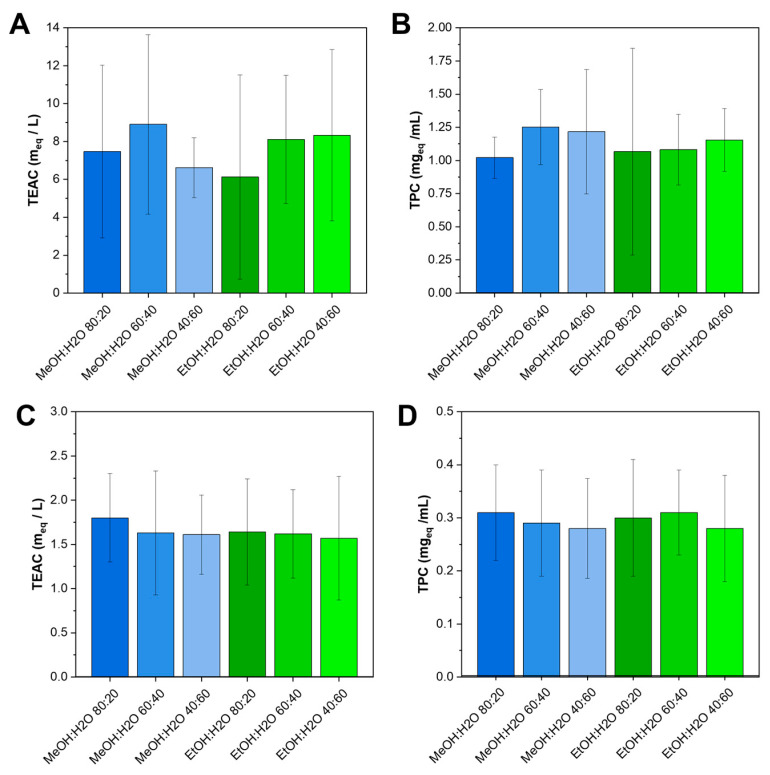
TEAC and TPC of turnip greens (**A**,**B**) leaf and (**C**,**D**) stem extracts, respectively, at different solvent compositions (namely MeOH/H_2_O and EtOH/H_2_O) and alcohol/water *v*/*v* ratios. Data are shown as mean ± 95% confidence interval (*n* = 3).

The selection of experimental parameters for investigation through the DoE was guided by the dual objective of achieving high extraction performances while ensuring process sustainability. In detail, traditional methods aimed to extract phenolics, chlorophyll, and other antioxidant molecules from brassicas rely on the use of aqueous solutions of methanol and ethanol at different percentages [22,27,28,29]. Based on these considerations, the same solutions were tested, as well as more dilute ethanol solutions (60 and 40%), in an attempt to develop a more environmentally sustainable process. Preliminary data collected on leaves and stems of turnip greens using different solvent compositions (Figure 2) show no significant differences (*p* > 0.05) resulting from the substitution of MeOH with EtOH in terms of TEAC and TPC of the extracts. These outcomes supported the choice of EtOH as the greener solvent and indicated that investigating higher alcohol levels was unnecessary. Therefore, methanolic solutions and concentrated ethanolic solutions (80%) were not further considered, while the solvent composition with an ethanol/water ratio ranging between 40 and 60% *v*/*v* was better investigated through the DoE approach. Furthermore, as previously reported in other studies [22,30], these data suggest that extracts obtained from leaves are richer in antioxidant compounds than those obtained from stems. Leaves, indeed, generally contain higher levels of polyphenols, flavonoids, and vitamin C, as they are metabolically more active tissues and are directly exposed to light and environmental stresses, which stimulate the synthesis of protective metabolites with antioxidant activity. In contrast, stems mainly serve structural and transport functions and are richer in fiber.

Regarding the other variables to be investigated through the DoE approach, extraction times equal to 5 and 15 min were selected based on preliminary experiments carried out by varying the time from 24 h to 1–2 h, down to a few minutes. An s/m value of 20 mL/g, suggested in several studies [29,31], was selected together with an s/m value of 10 in an attempt to make the process more sustainable. In addition, the application of ultrasound, in comparison with duration-matched SMs, was included among the process variables to evaluate its potential benefits. Finally, it was decided to operate at room temperature with the aim of reducing energy consumption while maintaining satisfactory extraction efficiency. The same DoE was conducted in parallel to investigate the extraction yield from leaves and stems separately since optimal processing conditions might differ for the two fractions. Extraction modality and time were merged into a four-level qualitative variable, including SMs for 5 min (SM 5′) or 15 min (SM 15′ or x_1a_) and UAEs for 5 min (UAE 5′% or x_1b_) or 15 min (UAE 15′ or x_1c_). Following the rules of thumb for the dummy coding [32], one level was selected as an implicit level to compare alternative conditions against such a reference. It was decided to adopt SM 5′ as an implicit level, being the most practical extraction option. A summary overview of the investigated process variables is provided in Table 2.

After adding three replicates (labeled as r1, r2, and r3) to the 16-experiment set, the variables were coded, and the extraction order was randomized, as reported in Table 3.

**Figure 3:**
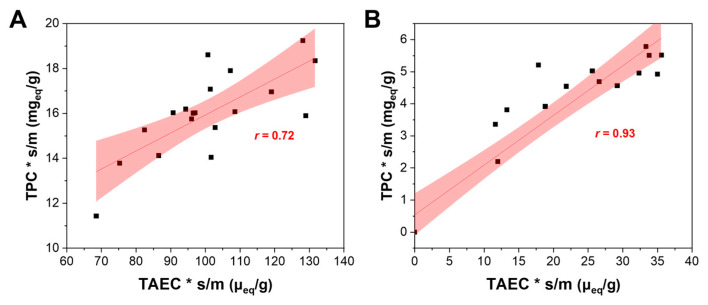
Correlation between TPC and TEAC responses in (**A**) leaves and (**B**) stems. Responses were multiplied by the s/m to compensate for the dilution effect it induced. The linear regression curves and their confidence intervals (95%) are shown in red.

A positive correlation emerged between TEAC and TPC measurements either on leaves (*r* = 0.72) or on stems (*r* = 0.93) (Figure 3). In fact, most of the extracted antioxidants are likely polyphenols and thus detectable by both assays. On the other hand, no such strong correlations were observed between stems and leaves when considering the same assay (*r* = 0.2 and 0.4 for TEAC and TPC, respectively, Figure S1). Evidently, leaves and stems have different chemical and physical characteristics, thereby justifying their separate analyses.

**Figure 4:**
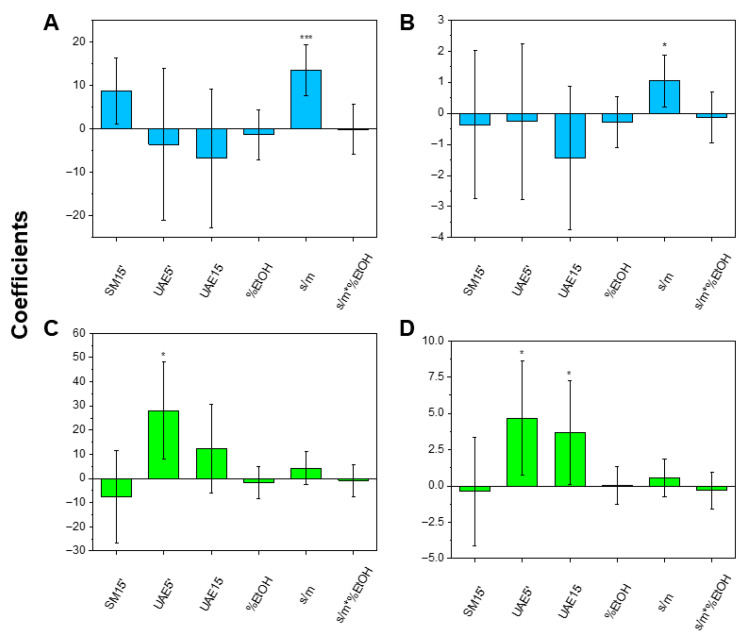
Effects of the studied variables on the extraction computed from the design of the experiment for leaves (panels **A**,**B**) and stems (panels **C**,**D**), as measured by the TEAC (left panels) and the TPC assays (right panels). Asterisks represent the statistical significance (* = *p*-value < 0.05; *** = *p*-value < 0.001).

Figure 4 and Table 4 show that the s/m value has a positive effect only for the leaves (panels A and B), while it is not significant for the stems (panels C and D). Additionally, leaves systematically exhibited higher responses than stems in both TEAC and TPC. This is likely due to a lower polyphenol content in the stems, for which even the low s/m level (*viz.*, 10 mL/g) is sufficient to extract the available polyphenolic fraction. However, despite the positive effect of s/m for leaves, exploring higher ratios would result in increased solvent consumption and elevated costs, ignoring the green chemistry principles. Because no significant differences were observed between the extraction modalities for leaves, SM 5′ was selected for this fraction as the most practical option. In contrast, the UAE outperformed SM for stems. Since extending the UAE duration to 15 min did not yield additional improvements, UAE 5′ was singled out for stems. Finally, %EtOH was not found to be significant in any case, allowing for operation at the low %EtOH level (*viz*. 40%). Whether assisted by ultrasounds or not, upon setting extraction durations at 5 min, any additional gains were likely to be negligible. To summarize the outcomes of this screening DoE, Table 5 reports the extraction conditions chosen for leaves (row 1) and stems (row 3).

For clarity, the extraction parameters resulting from the DoE are indicated in bold in Table 5. The other parameters, as mentioned above, were chosen based on time savings and solvent reduction, as they were not decisive for improving the extraction compared to the outputs considered (TEAC and TPC). However, it is conceivable that the phenolic acid profile could also be influenced by these latter extraction parameters. Similarly, these parameters could also affect the extraction of other molecules from the turnip greens waste with interesting biological activity. For this reason, the chromatographic analysis and the antimicrobial tests were extended to two other extracts in addition to those already optimized, one from the leaves and one from the stems, by randomly varying one extraction parameter from those previously arbitrarily chosen (Table 5, rows 2 and 4). In this way, the subsequent characterization and experiments were conducted on a total of 4 extracts (Table 5, last column).

### 2.3. Phenolic Acid Profile

To characterize the phenolic acid composition of the extracts, aliquots were subjected to complete hydrolysis to determine the total extractable phenolic acid content (i.e., free plus conjugated forms that were solubilized during ethanol extraction). This analysis was performed solely for compositional profiling; the extracts used in biological assays (Section 2.4) were not subjected to hydrolysis and contained phenolic acids in their native forms (free acids, glycosides, and soluble esters). The phenolic acid profiles of extracts obtained under the conditions reported in Table 5 are shown in Table 6. Caffeic, *p*-coumaric, ferulic, and sinapic acids were identified in both leaf extracts (L-SM and L-UAE). Compared to conventional silent maceration (L-SM), the ultrasound-assisted extract (L-UAE) showed a marked increase in total extractable caffeic and sinapic acid contents, which more than doubled (from 288 to 565 μg/g and from 412 to 1070 μg/g, respectively), while *p*-coumaric and ferulic acid contents remained relatively unchanged. The selective enhancement of caffeic and sinapic acid extraction by UAE can be explained by several interconnected mechanisms documented in the literature on ultrasound-assisted extraction from plant matrices. First, the mechanical effects of ultrasonic cavitation—including cell wall disruption, increased solvent penetration, and enhanced mass transfer—are particularly beneficial for extracting phenolic acids that exist predominantly in conjugated forms (esters and glycosides) within the plant cell walls [33,34]. Studies on *Brassica oleracea* var. *sabellica* (kale) demonstrated that ultrasound treatment significantly influences the recovery of hydroxycinnamic acids depending on solvent composition and extraction time, with each phenolic acid showing distinct optimal conditions [29]. Second, the UAE may facilitate partial in situ hydrolysis of labile ester linkages during the extraction process itself, particularly for compounds such as chlorogenic acids (caffeoyl-quinic acid esters) and sinapoyl-malate, which are abundant in Brassicaceae leaves [35,36]. The cavitational forces generated by ultrasound can promote the cleavage of these ester bonds, thereby increasing the amount of free phenolic acids recovered after subsequent complete hydrolysis and analysis. Third, the differential chemical stability of phenolic acids under ultrasonic conditions plays a role: while *p*-coumaric and ferulic acids are relatively stable in aqueous ethanol solutions under ultrasound, caffeic and sinapic acids can undergo sonochemical reactions in model systems [37]. However, in complex plant matrices, the beneficial extraction effects outweigh potential degradation, particularly at the moderate conditions employed in this study (5 min, room temperature, 40% ethanol). Our results indicate that caffeic and sinapic acid derivatives in turnip green leaves were more strongly bound within the cell wall matrix and therefore benefited disproportionately from the enhanced extraction efficiency provided by UAE, while *p*-coumaric and ferulic acid forms were already efficiently extracted by conventional maceration. In contrast, stem extracts (S-40 and S-60) contained considerably lower amounts of phenolic acids, with *p*-coumaric, ferulic, and sinapic acids detected only in trace amounts. The total extractable phenolic acid content in leaf extracts was consistent with the significantly higher TPC values observed for leaves compared to stems (Table 3), confirming that photosynthetically active tissues accumulate significantly greater amounts of these secondary metabolites [22,38]. Quantitative comparison with other Brassicaceae species provides further context for our results. Rodríguez García and Raghavan [22] reported that microwave-assisted extraction from broccoli (*B. oleracea* var. *italica*) yielded substantially higher total phenolic content in leaves (1940 μg GAE/g DW) compared to stems (225 μg GAE/g DW)—an 8.6-fold difference. Their HPLC analysis identified the same four major phenolic acids we detected (caffeic, *p*-coumaric, ferulic, and sinapic), with sinapic acid being particularly abundant in the leaf extracts. Similarly, Francisco et al. [39] found that in the *B. rapa* group—the same species as our turnip greens—total phenolic compounds were significantly higher in leaves harvested during the vegetative period (51.71 μmol/g DW, equivalent to approximately 8.8 mg GAE/g) compared to stems with flower buds and surrounding leaves (38.99 μmol/g DW, approximately 6.6 mg GAE/g). Our measured TPC values for leaves (14–19 mg GAE/g DW) fall within the upper range reported for *Brassica* vegetables and reflect the efficacy of our optimized extraction conditions. The marked difference between leaves and stems observed in our study—with stems showing near-trace levels of individual phenolic acids—is consistent with the physiological distribution of these compounds, as photosynthetically active leaf tissues require higher concentrations of phenolic antioxidants for protection against oxidative stress and UV radiation [38]. The phenolic acids characterized represent approximately 13–15% of the total measured TPC in leaf extracts; therefore, the selective increase induced by UAE on the concentrations of caffeic and sinapic acid does not contradict the conclusions of the DoE (according to which the extraction method is not a determining parameter for the improvement of TEAC and TPC). The remaining phenolic fraction likely comprises flavonoid glycosides (particularly kaempferol and quercetin derivatives) and hydroxycinnamic acid conjugates (e.g., sinapoyl-malate, caffeoyl-quinic acids), which are the predominant phenolic compounds in Brassicaceae and are efficiently extracted by aqueous ethanol [36,40]. In the non-hydrolyzed extracts used for biological assays, these phenolic acids exist in various forms—free acids, glycosides, and soluble esters—which may exhibit different bioavailability and biological activities. The observed antibiofilm efficacy (Section 2.4) is therefore attributed to the combined action of these native phenolic acid derivatives, along with other polyphenolic compounds contributing to the measured TPC values. Beyond phenolic compounds, Brassicaceae are also characterized by glucosinolates—sulfur-containing secondary metabolites structurally distinct from phenolics. While these compounds were not measured in the present study, it is important to clarify that intact glucosinolates do not contribute to the measured TPC values (as they are not detected by the Folin–Ciocalteu assay), nor to the observed TEAC [41] and antibiofilm activities [42]. In aqueous ethanol extracts such as those employed in this study, glucosinolates are likely present in their intact form. The presence of ethanol, even at moderate concentrations (40–60%), is known to inactivate myrosinase, the enzyme responsible for glucosinolate hydrolysis, thereby preventing the formation of bioactive breakdown products during extraction [43,44]. Intact glucosinolates exhibit limited biological activity; the health-promoting properties attributed to this class of compounds—including anticancer, antimicrobial, and antioxidant activities—are primarily associated with their enzymatic hydrolysis products, particularly isothiocyanates [42,43]. Future research could explore integrated valorization strategies employing sequential or controlled enzymatic hydrolysis in aqueous buffer systems to simultaneously recover phenolic compounds via solvent extraction and bioactive isothiocyanates via myrosinase activation, thereby maximizing the functional value of turnip green waste.

### 2.4. Biofilm-Inhibitory Activity

Some antioxidant molecules contained in vegetables of the broccoli group, such as flavonoids and polyphenols, not only have the ability to support the immune system’s response to infection but also have intrinsic antimicrobial activity. These effects may help limit foodborne pathogens such as *E. coli*, *Salmonella* spp., and *L. monocytogenes*, offering potential benefits amid rising antibiotic resistance [45]. In our experiments, four extracts obtained from broccoli leaves and stems (see Table 5) were tested against six pathogenic strains: *A. baumannii*, *E. coli*, *K. pneumoniae*, *L. monocytogenes*, *P. aeruginosa*, and *S. aureus*, some of whom belong to the so-called “ESKAPE” group, responsible for the majority of nosocomial infections and aptly named so also for their ability to ‘escape’ the lethal effects of antibiotics and disinfectants [46].

**Figure 5:**
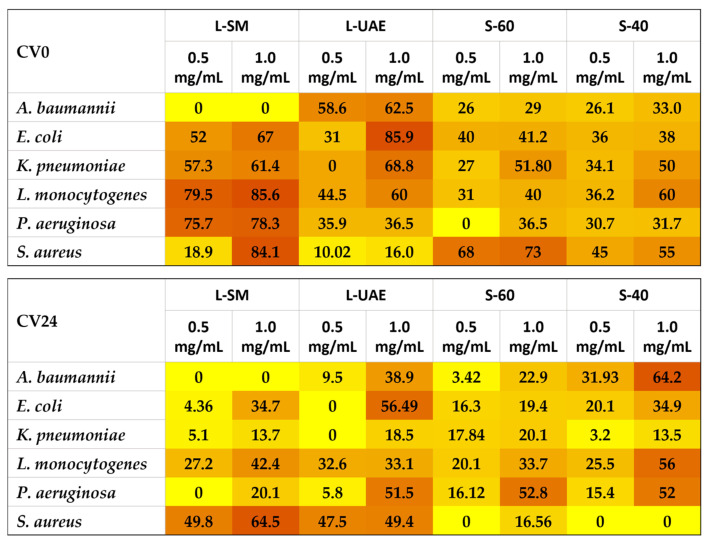
Heat map showing the inhibitory activity of the extracts, evaluated by the crystal violet (CV) test, against the microbial biofilm formation (CV0) and against the mature biofilm (CV24). The results are presented as percentages, assuming for the control (untreated bacteria) an inhibition = 0. The darker the color, the more potent the inhibitory activity. Sample concentration tested: 0.5 and 1.0 mg/mL.

To assess the inhibitory efficacy of the extracts against pathogenic biofilms, a minimum inhibitory concentration (MIC) test was performed first. Since the MIC values for all six pathogens were greater than 1.25 mg/mL (see Table S1), two concentrations (0.5 and 1 mg/mL) of the extracts were selected for subsequent assays. The biofilm-inhibitory effect of the extracts was investigated through the crystal violet test at two distinct stages: during biofilm formation (indicated in Table S2 and Figure 5 as CV0) and after the biofilm was fully established (mature biofilm, indicated in Table S2 and Figure 5 as CV24). The results reported for CV0 show that the two extracts obtained from broccoli leaves exhibited, in some cases, diverse behaviors compared with those derived from stems. In general, the leaf extracts (L-SM and L-UAE, obtained by SM or UAE, respectively, in 5 min with EtOH 40%) demonstrated a remarkable ability to prevent biofilm formation ab origine. This was particularly evident against *E. coli*, where an inhibitory effect of up to 85.9% was observed in the presence of the L-UAE extract, as well as against *L. monocytogenes*, with inhibition values of 85.6% caused by the L-SM extract. The inhibitory effect detected is significant not only from a food safety perspective but also from a health standpoint. Extracts obtained from broccoli leaf—an agro-industrial by-product—through a sustainable process could be used to produce mixtures of bioactive compounds suitable for industrial applications, for instance, in the food sector, as functional components of packaging materials [47]. In general, almost all leaf-derived extracts at the highest tested concentration inhibited the biofilm formation, never falling below 16.0% (sample L-UAE against *S. aureus*). Extracts obtained from the stem (S-60 and S-40, obtained by UAE in five minutes with EtOH 60% and 40%, respectively) generally showed slightly lower efficacy. However, in some cases (as observed in the test carried out with S-40), we noticed an inhibitory capacity of 60% against *L. monocytogenes*. Our results are partially in agreement with Hu et al. (2019), who demonstrated a weak inhibitory effect of stem extracts from different Brassicaceae on *E. coli* biofilm [48]. However, their work did not include broccoli. Overall, *S. aureus* was more sensitive than the other pathogens to the stem extracts, which showed biofilm-inhibitory effects ranging from 45% (sample S-40 at 0.5 mg/mL) to 73% (S-60 at 1 mg/mL).

In a subsequent phase, as described in the Section 3, the extracts were added to the bacterial cultures 24 h after the onset of growth. Thus, our study aimed to evaluate the effect of the extracts not only during biofilm formation but also on mature biofilms. Investigating both stages is crucial, as it might provide insights into strategies to prevent biofilm development ab origine and to fight mature biofilm, in which sessile cells become far more resistant and aggressive than planktonic cells from free-living cultures. The results, reported in Table S2 and Figure 5 as CV24, indicate that mature biofilms were generally more resistant to the extracts than the immature forms. Leaf-derived extracts retained appreciable antibiofilm activity against *S. aureus*, with inhibitory effects of 64.5% for L-SM at the highest concentration tested. Interestingly, while the mature biofilm of *S. aureus* was sensitive to almost all the leaf-derived extracts, it appeared almost entirely resistant to all the stem-derived extracts, regardless of the extraction method employed, except when tested against S-60 (inhibition = 16.56%). Stem-derived extracts retained inhibitory activity against the mature biofilm of the remaining five pathogenic strains, with inhibition values reaching 52% (S-40 against *P. aeruginosa*), 56% (S-40 against *L. monocytogenes*), and up to 64.2% (S-40 against *A. baumannii*), respectively. These observations underscore the importance of evaluating the activity across both Gram-positive and Gram-negative species, which can exhibit diverse resistance profiles [49,50]. Comparing the behavior of the extracts against the immature and mature biofilms, it is noteworthy that, in some cases—such as when we tested S-40 against *A. baumannii*—the inhibitory efficacy of such extract increased from 33.0% to 64.2%, suggesting that this extract could represent a promising candidate both to prevent biofilm formation and to act against mature biofilms of this pathogen, as well as those of *L. monocytogenes* and *P. aeruginosa*.

**Figure 6:**
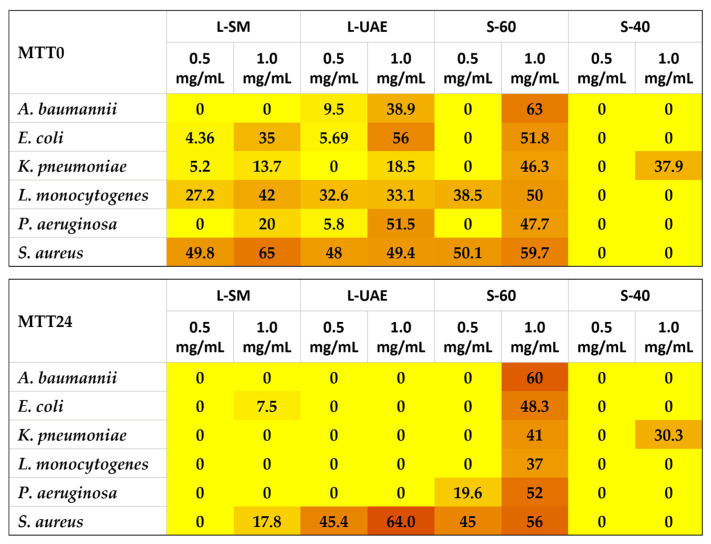
Heat map showing the inhibitory activity of the extracts, evaluated by the MTT test, against the metabolism of sessile cells present within the immature biofilm (MTT0) and mature biofilm (MTT24). The results are presented as percentages, assuming for the control (untreated bacteria) an inhibition = 0. The darker the color, the more potent the inhibitory activity. Sample concentration tested: 0.5 and 1.0 mg/mL.

The MTT assay, conducted in parallel with the CV test (Table S3 and Figure 6), allowed us to assess whether the efficacy of the extracts could be attributed, at least in part, to their direct action on the metabolic pathways of sessile cells within the biofilm, both during its formation (MTT0) and in the mature stage (MTT24). This investigation is particularly relevant, as the biochemical changes accompanying the transition to the sessile state render the cells markedly more aggressive than their planktonic counterparts, thereby contributing, through metabolic alterations within the biofilm, to its increased resistance to the conventional antibiotics [51]. The results of the MTT assay performed on immature biofilms show that, for samples L-SM and L-UAE (leaf-derived extracts), a consistent correlation between the inhibitory effects on biofilm formation and on sessile-cell metabolism was not always observed. However, despite some exceptions (including cases of complete inefficacy), the leaf extracts generally exerted a detectable effect on the metabolism of sessile cells within the developing biofilm.

As regards the samples obtained from the stems, some correspondence in action was not always observed, indicating that the inhibitory effect on biofilm formation induced by the samples could not be due only to a metabolic inhibitory action on the sessile cells. However, extract S-60 showed a decisive inhibitory effect on sessile-cell metabolism (63%) against *A. baumannii* and a substantial increase in inhibitory activity against almost all bacterial strains compared to the respective inhibitory results of the CV assay.

Therefore, we agree with the hypothesis supported by other studies [52], which found that the extracts acting on the biofilm can, but not always, inhibit the metabolic pathway. On the other hand, these findings suggest that the inhibitory action of our extracts primarily can result from interference with the molecular mechanisms driving biochemical transitions in sessile cells, thereby preventing their shift toward more aggressive and difficult-to-eradicate phenotypes. Indeed, a lack of linear correlation between biofilm-inhibitory efficacy and the ability to reduce the metabolic activity of sessile cells has been reported in several studies. However, these studies were performed on different matrices. This discrepancy likely reflects the complexity of biofilm response mechanisms, in which biomass reduction does not necessarily coincide with a parallel impairment of cellular viability or intracellular metabolic pathways [50,51,53,54].

The results of the MTT assay performed on mature biofilms (MTT24, in Table S3 and Figure 6) clearly show that leaf extracts are ineffective in inhibiting the metabolism of sessile cells. A notable exception was observed for *S. aureus*, where all leaf-derived extracts displayed measurable inhibition, sometimes exceeding that observed in the MTT assay of immature biofilms. For instance, L-UAE increased its inhibitory activity from 49.4% to 64.0%. Among the stem-derived extracts, sample S-60 tested at the highest concentration proved to be the most effective in inhibiting mature biofilms of the various strains tested, showing the strongest inhibitory activity (60%) against *A. baumannii.* The distinct behavior of leaf- and stem-derived extracts highlights that their different qualitative and quantitative compositions may influence this biological property.

Overall, the results indicate that broccoli extracts can inhibit biofilm formation and mature biofilm through mechanisms beyond biomass reduction alone. These may include modulation of biochemical pathways within sessile cells, such as those involved in cell adhesion, intercellular communication, and responses to environmental stress. The observation that biofilm-inhibitory activity does not always correlate with reduced sessile-cell metabolism underscores the need for further studies to identify which molecular pathways these extracts specifically target and to explore their biotechnological and therapeutic potential.

## 3. Materials and Methods

### 3.1. Reagents

Absolute ethanol, methanol, 2,2′-azino-bis(3-ethylbenzothiazoline-6-sulfonic acid) (ABTS), 6-hydroxy-2,5,7,8-tetramethylchroman-2-carboxylic acid (Trolox), potassium persulfate, Folin–Ciocalteu reagent, anhydrous sodium carbonate, gallic acid, and ethyl acetate were obtained from Merck Italy (Merck Life Science s.r.l., Milan, Italy). Bacterial growth media broth, tetracycline, dimethylsulfoxide, resazurin, crystal violet (CV), and 3-[4,5-dimethylthiazol-2-yl]-2,5 diphenyl tetrazolium bromide (MTT) were provided by Sigma Aldrich, Milan, Italy. All chemicals used were of analytical grade and were used as received without any further purification.

### 3.2. Sample Preparation and Characterization

The stems and leaves of turnip greens (*Brassica rapa* subsp. *Sylvestris*, Accession Numbers: 303239 of Mediterranean Germplasm Database) were collected from a local market in Bari, Apulia region, Italy. The leaves and stems were first separated and then cut into small pieces (approximately 5 cm) and finally aliquoted in vacuum-packed plastic bags (500 g for each category). The resulting material was immediately frozen (−80 °C) and subsequently freeze-dried (Heto LyoLab 3000, Thermo Scientific, Waltham, MA, USA). Before extraction, the freeze-dried leaves and stems were ground into a powder, first using a commercial grinder and then using an agate mortar. A particle size analysis was conducted on the powders using calibrated sieves (Test Sieve, Endecotts Limited, Lombard Road, London, UK). Finally, the powders were stored at −20 °C until the extraction (see Section 3.4).

### 3.3. Design of Experiment

A DoE approach was employed to investigate the extraction conditions. Data analysis was performed with the CAT (Chemometric Agile Tool) software [55], developed in the R environment (version 3.1.2). The parameters considered included extraction time, solvent-to-matrix ratio (s/m), solvent composition, and extraction mode (SM versus UAE). To limit the total experimental effort, extraction mode and duration were combined into a single categorical variable (named “APPROACH”) with four categorical levels. A five-minute SM was set as an implicit level.

Extraction efficiency was separately assessed for each plant fraction (namely, leaves and stems) using both total phenolic content (TPC) by the Folin–Ciocâlteu assay and Trolox equivalent antioxidant capacity (TEAC) by the ABTS assay. Based on the number of factors and their levels, four multiple linear regression models were obtained, each comprising 7 coefficients (1 constant, 5 linear terms, and 1 interaction), as represented by the following equation:

(1) $$y=\;b_{0}+\;b_{1a}x_{1a\;}+\;b_{1b}x_{1b\;}+\;b_{1c}x_{1c\;}+\;b_{2}x_{2}+b_{3}x_{3}+b_{23}x_{2}x_{3}+\varepsilon$$

For each plant fraction, three experimental replicates were selected from the proposed experimental matrix through the D-optimal by addition strategy and incorporated into the experimental set to evaluate the experimental variability of the process.

### 3.4. Polyphenol Extraction

#### 3.4.1. Ultrasound-Assisted Extraction

1 g of ground leaves or stems was placed in a conical flask equipped with a stopper, and a specific amount of extraction mixture was added. The system was then placed in an ultrasonic bath (Elmasonic S100H, Elma Schmidbauer GmbH, Singen, Germany) operating at 550 W and 50/60 Hz at room temperature (RT) for the selected time. To stop the extraction, the mixture was centrifuged at 4 °C for 10 min at 6000 rpm. The centrifugation time was considered an integral part of the overall extraction process, kept constant in all experiments, and was not taken into account as a DoE variable. The supernatant was collected and centrifuged again to remove any plant residues. Finally, the extract obtained was filtered using disposable syringe filters with a porosity of 0.20 µm and stored at −20 °C until further analysis.

#### 3.4.2. Silent Maceration

1 g of ground leaves or stems was placed in a bottle equipped with a stopper, and a specific amount of extraction mixture was added. The system was left under stirring (100 rpm) for a specific amount of time at RT. The final extract was recovered as described in Section 3.4.1.

### 3.5. Determination of TPC

The TPC of the extracts was determined according to the Folin–Ciocalteau method with some modifications [56]. To this end, 800 µL of water, 50 µL of the sample suitably diluted in H_2_O, and 50 µL of Folin–Ciocalteu reagent were added to conical test tubes. Within 8 min of the addition of the reagent, 100 µL of a 20% *w*/*w* aqueous solution of Na_2_CO_3_ was added. For the baseline, 50 μL of water was added instead of the sample. The tubes were incubated in the dark for 2 h at RT. Following incubation, the solutions took on a typical blue color, indicating that the polyphenols in the sample had been oxidized by the reagent. The absorbance of each sample was measured at 760 nm. The TPC was expressed in terms of mg of gallic acid (GA) equivalents, for which a calibration curve was constructed, and subsequently multiplied by the s/m value and expressed as mg_eq_ GA/g of extracted plant material. The analysis was performed in triplicate.

### 3.6. Antioxidant Activity

The antioxidant activity of the extracts was evaluated through the ABTS colorimetric assay. For this purpose, a solution of 0.008 g/mL ABTS and a solution of 0.0132 g/mL K_2_S_2_O_8_ in distilled water were prepared. Then 1 mL of each solution was mixed and left to react for at least 16 h in dark conditions. The solution, initially colorless, turns deep blue following the oxidation of ABTS and, therefore, the formation of radical species. For the test, the ABTS^•+^ solution was diluted with ethanol until reaching an absorbance value at 750 nm between 0.7 and 1.0. Thus, 50 µL of properly ethanol-diluted vegetal extract was added to 950 µL of diluted ABTS^•+^ and allowed to react in the dark for 30 min at 25 °C under shaking at 40 rpm (SKI 4 shaker incubator). After incubation, the absorbance of each sample was evaluated at 750 nm. The antioxidant activity of each extract was calculated in terms of TEAC after constructing a specific calibration curve and then multiplying by the s/m value and expressed as μ_eq_ of Trolox/g of plant material used for extraction. The analysis was carried out in triplicate.

### 3.7. Phenolic Acids Characterization

To determine the total extractable phenolic acid content, aliquots of the dried extracts were subjected to complete hydrolysis prior to High-Performance Liquid Chromatography (HPLC) analysis to release phenolic acids from soluble conjugated forms (glycosides and esters). Phenolic acids were extracted from 0.25 g of dried extract powder (obtained as described in Section 3.4.1 and Section 3.4.2) following sequential alkaline and acidic hydrolysis according to [57]. Alkaline hydrolysis was performed by incubating samples with 2 M NaOH at 4 °C for 1 h in the dark to minimize oxidative degradation of phenolic acids. Following acidification to pH 2 with HCl, the hydrolysates were immediately extracted with ethyl acetate, dried under a nitrogen stream, redissolved in 100 μL of 80:20 (*v*/*v*) methanol/water, and analyzed by HPLC. HPLC analysis was carried out using an Agilent 1100 Series HPLC with Diode Array Detection (DAD) system (Agilent Technologies, Santa Clara, CA, USA) equipped with a reversed-phase C18(2) Luna LC column (5 μm, 250 × 4.6 mm; Phenomenex, Torrance, CA, USA). Phenolic acids were separated as described in [58], identified by comparing retention times (within acceptable tolerance ranges) and UV-Vis spectral profiles with those of authentic standards using spectral matches.

### 3.8. Antibacterial and Antibiofilm Activity

#### 3.8.1. Antibacterial Test

Dried extracts from leaves and stems were accurately resuspended in sterile water. Their potential antibacterial activity was evaluated against both Gram-negative strains (*Acinetobacter baumannii* ATCC 19606, *Pseudomonas aeruginosa* DSM 50071, *Escherichia coli* DSM 8579, and a clinical isolate of *Klebsiella pneumoniae*, identified at the species level using the chromogenic medium Klebsiella ChromoSelect Selective Agar Base) and Gram-positive strains (*Listeria monocytogenes* ATCC 7644 and *Staphylococcus aureus* subsp. *aureus* ATCC 25923). Before testing, bacterial suspensions were cultured in Luria–Bertani (LB) broth for 18 h under continuous shaking at 80 rpm (Corning LSE incubator, Pisa, Italy). Incubation was carried out at 37 °C for all strains, except for *A. baumannii*, which was maintained at 35 °C. The minimal resazurin microtiter plate assay was employed to determine the minimum inhibitory concentration (MIC), according to the protocols described by Fratianni et al. [50] and Sarker et al. [59]. The antibacterial assay was performed in flat-bottom 96-well microplates using different concentrations of the extracts, previously diluted in sterile deionized water, and incubated for 24 h at 35–37 °C, depending on the strain. Sterile dimethyl sulfoxide (DMSO) was used as the negative control and tetracycline (1 mg/mL in DMSO) as the positive control.

#### 3.8.2. Effect of the Extracts on Biofilm Formation

The ability of the extracts to inhibit initial bacterial adhesion was evaluated according to the protocol described by Fratianni et al. [50], using flat-bottom 96-well microtiter plates. Overnight bacterial cultures were adjusted to the 0.5 McFarland standard (approximately 1.5 × 10^7^ cells/mL) with fresh LB broth. Each well received 10 μL of bacterial suspension, the extracts at two final concentrations (40 or 80 μL/mL), and LB broth, for a total volume of 250 μL. The plates were sealed with parafilm to prevent evaporation and incubated for 48 h at 35 °C or 37 °C (depending on the strain). After incubation, non-adherent cells were discarded, and the wells were gently washed twice with sterile phosphate-buffered saline (PBS). Adherent cells were fixed with 200 μL of methanol for 15 min, stained with 2% crystal violet for 20 min, rinsed with PBS, and air-dried. The bound dye was then solubilized with 200 μL of 20% glacial acetic acid, and absorbance was measured at 540 nm. The percentage inhibition of adhesion was calculated relative to the untreated control (considered as 0% inhibition).

#### 3.8.3. Effect of the Extracts on Mature Biofilm

The experimental procedure was conducted following the protocol described by Fratianni et al. [50]. Overnight bacterial cultures were adjusted to the 0.5 McFarland standard using fresh LB broth, and 10 μL of each suspension was dispensed into flat-bottom 96-well microplates, resulting in a final volume of 250 μL per well. The microplates were sealed with parafilm to prevent evaporation and incubated at 35 °C or 37 °C (depending on the strain) for 24 h. Then, planktonic cells were carefully removed. The extracts were tested at final concentrations of 40 μL/mL and 80 μL/mL in LB broth, with a total volume of 250 μL per well. The plates were subsequently incubated for an additional 24 h. After incubation, non-adherent cells were discarded, and the wells were gently washed twice with sterile PBS. Adherent cells were fixed with 200 μL of methanol for 15 min, stained with 2% crystal violet for 20 min, rinsed with PBS, and air-dried. The bound dye was then solubilized with 200 μL of 20% glacial acetic acid, and absorbance was measured at 540 nm. The percentage of inhibition was calculated relative to the untreated control (bacterial cultures grown in the absence of the extracts, considered 0% inhibition).

#### 3.8.4. Effect of the Extracts on the Metabolic Activity of Sessile Cells

The effect of the extracts on the metabolic activity of biofilm-embedded bacterial sessile cells was assessed using the 3-(4,5-dimethylthiazol-2-yl)-2,5-diphenyltetrazolium bromide (MTT) colorimetric assay, following the established procedure [50,60]. Two concentrations (40 μL/mL and 80 μL/mL) were tested by adding the extracts either at the beginning of bacterial growth or after 24 h of incubation. After a total incubation period of 48 h, the planktonic cell suspension was carefully removed, and 150 μL of sterile PBS together with 30 μL of 0.3% MTT solution (Sigma, Milan, Italy) were added to each well. The microplates were then incubated at 37 °C or 35 °C (depending on the strain) for two hours. Subsequently, the MTT solution was removed, and the wells were washed twice with 200 μL of sterile physiological solution. Formazan crystals were solubilized by adding 200 μL of DMSO, and the absorbance was measured at 570 nm (Cary Varian, Milan, Italy) after one hour. The inhibitory percent value for the sessile cells’ metabolism was calculated relative to the control (bacterial cells grown without the presence of the samples), for which the inhibition rate was assumed to be 0%.

### 3.9. Statistical Analysis

Preliminary comparisons between extraction methods were performed in triplicate. Pearson correlation coefficient (*r*), mean, standard deviation (SD), and confidence interval (CI) were computed through “Excel Statistics” version 365.

Microbial experiments were performed in triplicate. Results were expressed as the mean ± SD of three experiments (PC software “Excel Statistics” version 365). Statistical significance was assessed by one-way ANOVA followed by Tukey’s HSD post hoc test and by one-way ANOVA followed by Dunnett (*p* < 0.05; *n* = 3).

## 4. Conclusions

The results of this study demonstrate that polyphenol-rich extracts can be sustainably obtained from green turnip byproducts by employing ethanol as a green solvent alternative to methanol and using the UAE technique instead of conventional maceration. Overall, extracts derived from turnip green leaves exhibited significantly higher total phenolic content and antioxidant activity compared to those obtained from the stems. HPLC analysis revealed the presence of several phenolic acids in the leaf extracts, including sinapic, *p*-coumaric, caffeic, and ferulic acids, whereas only negligible amounts were detected in the stem extracts. The antibacterial activity of extracts against biofilms formed by six pathogenic bacterial strains was assessed using crystal violet and MTT assays, indicating significant inhibitory effects on biofilm formation and mature biofilm stages. This data is particularly important for the bacteria we used in the tests, which, except for *Listeria monocytogenes*, belong to the so-called ESKAPE group, known for their high levels of multidrug resistance. Although further insights are necessary, the conducted study demonstrates the potential for valorizing turnip greens by-products both as health supplements and as preservatives in the food and packaging industry.

## Figures and Tables

**Table 1:** Characterization of shredded leaves and stems in terms of particle size fractions.

Leaves		Stems	
Diameter (mm)	*w*/*w* (%)	Diameter (mm)	*w*/*w* (%)
>2	0.27 ± 0.14	>2	3.1 ± 0.4
2 > d > 1	31 ± 5	2 > d > 1	28 ± 5
1 > d > 0.5	50 ± 4	1 > d > 0.5	41 ± 3
0.5 > d > 0.1	15 ± 3	0.5 > d > 0.1	24 ± 7

**Table 2:** Factors involved in the extraction process and their levels explored by the 2^k^ factorial design.

Variable Name	Var. ID	Type	Unit	Levels	
APPROACH ^a^	x_1_	Qualitative	–	SM 5′ (implicit)	SM 15′ ^a^
				UAE 5′	UAE 15′
%EtOH	x_2_	Quantitative	% (*v*/*v*)	40 (−1)	60 (+1)
s/m ^b^	x_3_	Quantitative	mL/g	10 (−1)	20 (+1)

^a^ Explicit levels of APPROACH (*viz.*, SM 15′, UAE 5′, and UAE 15′) are also labeled as x_1a_, x_1b_, and x_1c_, respectively; ^b^ s/m = solvent-to-matrix ratio.

**Table 3:** Experimental plan and experimental matrix along with the observed TEAC and TPC responses for both leaves and stems.

Run	Experimental Plan			Experimental Matrix					Response			
	x_1_	x_2_	x_3_	x_1a_	x_1b_	x_1c_	x_2_	x_3_	y ^a^			
									Leaves		Stems	
	Approach	%EtOH	s/m ^b^	SM 15′	UAE 5′	UAE 15′	%EtOH	s/m	TEAC ^d^	TPC ^e^	TEAC ^d^	TPC ^e^
1 r1	SM 15′	40	10	1	0	0	−1	−1	81.5	15.8	0 ^c^	0
2	UAE 5′	60	10	0	1	0	1	−1	96.5	16.0	53.2	9.4
3	UAE 5′	40	10	0	1	0	−1	−1	97.0	16.0	58.4	9.1
4	UAE 15′	40	20	0	0	1	−1	1	128.1	19.2	33.8	5.5
5	SM 5′	60	20	0	0	0	1	1	119.1	17.0	32.3	5.0
6 r1	SM 15′	40	10	1	0	0	−1	−1	101.7	14.1	0	0
7	SM 15′	60	20	1	0	0	1	1	129.0	15.9	17.8	5.2
8	SM 15′	60	10	1	0	0	1	−1	101.4	17.1	0	0
9	SM 15′	40	20	1	0	0	−1	1	131.7	18.3	25.6	5.0
10	UAE 15′	60	20	0	0	1	1	1	108.5	16.1	21.9	4.5
11 r2	UAE 15′	40	10	0	0	1	−1	−1	86.5	14.1	37.6	7.8
12	SM 5′	60	10	0	0	0	1	−1	90.7	16.0	0	0
13 r3	UAE 15′	60	10	0	0	1	1	−1	68.5	11.4	26.6	7.6
14	SM 5′	40	10	0	0	0	−1	−1	94.4	16.2	0	0
15	UAE 5′	40	20	0	1	0	−1	1	102.8	15.4	33.4	5.8
16	UAE 5′	60	20	0	1	0	1	1	100.8	18.6	35.0	4.9
17 r3	UAE 15′	60	10	0	0	1	1	−1	75.3	13.8	23.3	6.7
18 r2	UAE 15′	40	10	0	0	1	−1	−1	82.5	15.3	24.0	4.4
19	SM 5′	40	20	0	0	0	−1	1	107.2	18.0	35.6	5.5

^a^ Responses were multiplied by the s/m to compensate for the dilution effect it induced; ^b^ solvent-to-matrix ratio is reported in mL/g; ^c^ It was not possible to recover the extract from stems when the s/m was set at level −1; ^d^ TEAC is reported as μ_eq_/g; ^e^ TPC is reported as mg_eq_/g.

**Table 4:** Mathematical models for TEAC and TPC responses for leaves and stems, obtained by the application of the DoE. The corresponding multiple linear regression models are reported as y = b_0_ + b_SM 15′_x_SM 15′_ + b_UAE 5′_x_UAE 5′_ + b_UAE 15′_x_UAE 15′_ + b_%EtOH_x_%EtOH_ + b_s/m_x_s/m_ + b_%EtOH,s/m_x_%EtOH_x_s/m_, with the number of digits reflecting the uncertainty associated with each coefficient.

Response	Equation
TEAC-leaves	y = 103 (***) + 9∙SM 15′ − 4∙UAE 5′ − 7∙UAE 15′ − 1∙%EtOH + 13∙s/m (***) − 0∙%EtOH∙s/m
TPC-leaves	y = 16.8 (***) − 0∙SM 15′ − 0∙UAE 5′ − 1∙UAE 15′ − 0.3∙%EtOH + 1.0∙s/m (*) − 0.1∙%EtOH∙s/m
TEAC-stems	y = 17 (***) − 8∙SM 15′ + 28∙UAE 5′ (*) + 12∙UAE 15′ − 2∙%EtOH + 4∙s/m − 1∙%EtOH∙s/m
TPC-stems	y = 3 − 0∙SM 15′ + 5∙UAE 5′ (*) + 4∙UAE 15′ (*) + 0∙%EtOH + 1∙s/m − 0.3∙%EtOH∙s/m

Statistical significance is shown as: * = *p*-value < 0.05; *** = *p*-value < 0.001.

**Table 5:** Selected extraction conditions for leaves and stems.

Plant Fraction	Approach	s/m (mL/g)	EtOH (% *v*/*v*)	Sample Acronym
Leaves	SM 5′	**20**	40	L-SM
	UAE 5′	**20**	40	L-UAE
Stems	**UAE 5′**	10	40	S-40
	**UAE 5′**	10	60	S-60

Extraction parameters resulting effective from the DoE are indicated in bold. The other parameters were chosen to pursue the green chemistry principles (e.g., time savings and solvent reduction).

**Table 6:** Identification and quantification of phenolic acids in selected extracts carried out by HPLC analysis.

	Leaves		Stems	
Phenolic Acid (μg/g)	L-SM	L-UAE	S-40	S-60
Caffeic acid	288 ± 27	565 ± 58	ND	ND
*p*-coumaric acid	239 ± 24	218 ± 20	6.6 ± 0.5	ND
Ferulic acid	648 ± 55	685 ± 70	3.6 ± 0.4	ND
Sinapic acid	412 ± 40	1070 ± 90	7.5 ± 0.6	2.50 ± 0.14

Values represent phenolic acids that were solubilized during ethanol extraction and subsequently released from glycosides and soluble esters by hydrolysis. ND = not detected.

## Data Availability

The original contributions presented in this study are included in the article. Further inquiries can be directed to the corresponding author.

## Supplementary Materials

The following supporting information can be downloaded at: https://www.mdpi.com/article/10.3390/molecules31020388/s1. Figure S1: Correlation between responses in leaves and stems in terms of (A) TEAC and (B) TPC. Table S1: MIC of L-SM, L-UAE, S-60, and S-40 extracts (mg/mL).; Table S2: The inhibitory activity of the extracts evaluated by the crystal violet test on microbial biofilm formation (CV0) and mature biofilm (CV24); Table S3: Inhibitory activity of the extracts against the sessile cells metabolism in early biofilm (MTT0) and mature biofilm (MTT24).
