# Supplementary material for: Valorization of Turnip Greens (Brassica rapa subsp. sylvestris) Wastes: Investigation on the Sustainable Recovery of Bioactive Extracts with Antioxidant and Antibiofilm Properties
Source: Molecules. 2026 Jan 22;31(2):388. doi: 10.3390/molecules31020388 (PMC12844269; doi:10.3390/molecules31020388)
Supplement: Supplementary file 1 [file molecules-31-00388-s001.zip › molecules-4038695-supplementary.pdf]

Supplementary Materials

Valorization of Turnip Greens (*Brassica rapa* subsp. *sylvestris*) Wastes: Investigation on the Sustainable Recovery of Bioactive Extracts with Antioxidant and Antibiofilm Properties

Anna Maria Maurelli[1], Davide Coniglio[2], Francesco Milano[3], Sara Mancarella[3], Barbara Laddomada[4], Vincenzo De Leo[2,*], Francesco Longobardi[2], Francesca Coppola[5], Florinda Fratianni[6], Michelangelo Pascale[6], Filomena Nazzaro[6], and Lucia Catucci[2]

[1] Institute of Food Sciences, Italian National Research Council (CNR-ISA), URT-Bari, via Orabona 4, 70126 Bari, Italy; annamaria.maurelli@isa.cnr.it

[2] Department of Chemistry, University of Bari Aldo Moro, via Orabona 4, 70126 Bari, Italy; davide.coniglio@uniba.it (D.C.); francesco.longobardi@uniba.it (F.L.); lucia.catucci@uniba.it (L.C.)

[3] Institute of Sciences of Food Production, Italian National Research Council (CNR-ISPA), S.P. Lecce-Monteroni, I-73100 Lecce, Italy; francesco.milano@cnr.it (F.M.); saramancarella@cnr.it (S.M.)

[4] Institute of Sciences of Food Production, Italian National Research Council (CNR-ISPA), Via Amendola, 122/O - 70126 Bari, Italy; barbara.laddomada@cnr.it

[5] Department of Agricultural Sciences, University of Naples Federico II, Piazza Carlo di Borbone 1, Portici (NA) 80055, Italy mail: francesca.coppola2@unina.it

[6] Institute of Food Sciences, Italian National Research Council (CNR-ISA), Via Roma 64, 83100 Avellino, Italy mail florinda.fratianni@isa.cnr.it (F.F.); filomena.nazzaro@cnr.it (F.N.); michelangelo.pascale@cnr.it (M.P.)

*Correspondence: vincenzo.deleo@uniba.it

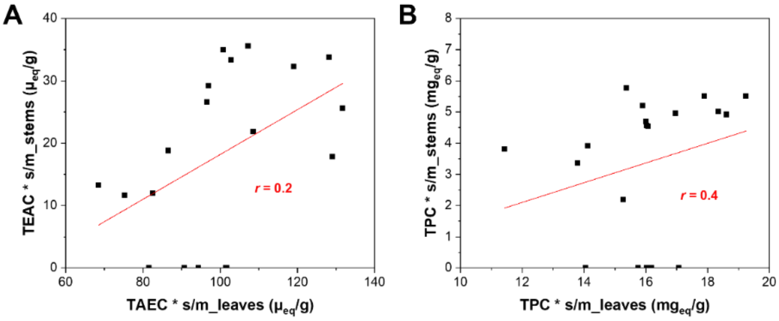

Figure S1. Correlation between responses in leaves and stems in terms (A) TEAC and (B) TPC.

Table S1. MIC of L-SM, L-UAE, S-60 and S-40 extracts (mg/mL).

Strain	L-SM	L-UAE	S-60	S-40	Tetracycline
A. baumannii	1.4 ± 0.1 ^{b}	1.4 ± 0.2 ^{b}	1.6 ± 0.2 ^{b}	1.4 ± 0.2 ^{b}	0.7 ± 0.1
E. coli	1.4 ± 0.2 ^{b}	1.35 ± 0.1^{b}	1.6 ± 0.2 ^{b}	1.4 ± 0.3 ^{b}	0.8 ± 0.2
K. pneumoniae	1.35 ± 0.1 ^{b}	1.5 ± 0.2 ^{b}	1.8 ± 0.2 ^{b}	1.4 ± 0.2 ^{b}	0.9 ± 0.2
L. monocytogenes	1.4 ± 0.3 ^{b}	1.4 ± 0.3 ^{b}	1.6 ± 0.2 ^{b}	1.35 ± 0.1	0.8 ± 0.2
P. aeruginosa	1.35 ± 0.1 ^{b}	1.6 ± 0.2 ^{b}	1.6 ± 0.3 ^{b}	1.4 ± 0.2 ^{b}	0.7 ± 0.02
S. aureus	1.4 ± 0.2 ^{b}	1.6 ± 0.3 ^{b}	1.6 ± 0.3 ^{b}	1.6 ± 0.3 ^{b}	0.8 ± 0.02

The results are presented as the mean (±SD) of three independent experiments. Tetracycline was used as the control. ^{b}significantly different from tetracycline (Dunnett test, $p < 0.05$)

Table S2. The inhibitory activity of the extracts evaluated by the crystal violet test on microbial biofilm formation (CV0) and mature biofilm (CV24).

**CV0**	**L-SM**		**L-UAE**		**S-60**		**S-40**	
	**0.5 mg/mL**	**1 mg/mL**	**0.5 mg/mL**	**1 mg/mL**	**0.5 mg/mL**	**1 mg/mL**	**0.5 mg/mL**	**1 mg/mL**
A. baumannii	0.0 ± 0.0	0.0 ± 0.0	58.6^{c} ± 0.7	62.5^{c} ± 0.6	25.7^{b} ± 1.1	29^{b} ± 2	26.1^{b} ± 1.7	33.0^{b} ± 1.6
E. coli	52^{b} ± 8	66.7^{c} ± 1.1	31^{b} ± 5	85.9^{d} ± 0.3	40^{b} ± 2	41.2^{b} ± 1.1	36^{b} ± 2	38^{b} ± 2
K. pneumoniae	57.3^{c} ± 0.5	61.4^{c} ± 1.4	0.00 ± 0.00	68.8^{c} ± 0.4	27^{b} ± 2	51.80^{b} ± 0.13	34.1^{b} ± 1.4	50^{b} ± 3
L. monocytogenes	79.5^{d} ± 1.5	85.6^{d} ± 0.4	44.5^{b} ± 1.4	60.0^{c} ± 1.0	31^{b} ± 3	40^{b} ± 2	36.2^{b} ± 1.8	60^{c} ± 3
P. aeruginosa	75.7^{d} ± 0.6	78.3^{d} ± 0.5	35.9^{b} ± 1.6	36.5^{b} ± 1.5	0.0 ± 0.0	36.5^{b} ± 1.5	30.7^{b} ± 1.0	31.7^{b} ± 1.1
S. aureus	18.9^{a} ± 1.7	84.1^{d} ± 0.3	10.02^{a} ± 0.08	16.0^{a} ± 1.0	68^{c} ± 2	73^{c} ± 2	45^{b} ± 3	55^{c} ± 2

**CV24**	**L-SM**		**L-UAE**		**S-60**		**S-40**	
	**0.5 mg/mL**	**1 mg/mL**	**0.5 mg/mL**	**1 mg/mL**	**0.5 mg/mL**	**1 mg/mL**	**0.5 mg/mL**	**1 mg/mL**
A. baumannii	0.0 ± 0.0	0.0 ± 0.0	9.5^{a} ± 0.8	38.9^{c} ± 0.5	3.42nd ± 0.09	22.9^{b} ± 0.7	31.9^{b} ± 1.1	64.2^{c} ± 0.7
E. coli	4.36nd ± 0.08	34.7^{b} ± 0.6	0.00 ± 0.00	56.49^{c} ± 0.04	16.3^{a} ± 0.6	19.4^{a} ± 1.3	20.1^{ab} ± 1.1	34.9^{b} ± 1.1
K. pneumoniae	5.1^{a} ± 0.5	13.7^{a} ± 0.5	0.00 ± 0.00	18.5^{a} ± 0.7	17.84^{a} ± 0.16	20.1^{b} ± 0.7	3.2nd ± 0.6	13.5^{a} ± 0.4
L. monocytogenes	27.2^{b} ± 0.7	42.4^{b} ± 0.6	32.6^{b} ± 0.7	33.1^{b} ± 0.7	20.1^{ab} ± 0.7	33.7^{b} ± 0.7	25.5^{b} ± 0.7	56^{bc} ± 0.7
P. aeruginosa	0.0 ± 0.0	20.1^{ab} ± 0.6	5.8^{a} ± 0.7	51.5 ± 0.4	16.12^{a} ± 0.18	52.8^{c} ± 0.4	15.4^{a} ± 1.1	52^{c} ± 1
S. aureus	49.8^{b} ± 0.5	64.5^{c}± 0.4	47.5^{b} ± 0.5	49.4^{b}± 0.5	0.00 ± 0.00	16.56^{a} ± 0.16	0.0 ± 0.0	0.0 ± 0.0

Results are presented as percentages, assuming for the control (untreated bacteria) an inhibition = 0. Sample concentration: 0.5 and 1 mg/mL. Samples: leaf-derived extract macerated for 5 minutes with EtOH 40% (L-SM); leaf-derived extract obtained by ultrasound treatment for 5' with EtOH 40% (L-UAE), stems-derived extract obtained by ultrasound treatment for 5" with EtOH 60% (S-60) and stems-derived extract obtained by ultrasound treatment for 5" with EtOH 40% (S-40). Note: equal letters denote no significant difference; different letters denote significant differences (one-way ANOVA + Tukey HSD, $p < 0.05$; n = 3).

Table S3. Inhibitory activity of the extracts against the sessile cells metabolism in early biofilm (MTT0) and mature biofilm (MTT24).

MTT0	L-SM		L-UAE		S-60		S-40	
	**0.5 mg/mL**	**1 mg/mL**	**0.5 mg/mL**	**1 mg/mL**	**0.5 mg/mL**	**1 mg/mL**	**0.5 mg/mL**	**1 mg/mL**
A. baumannii	0.0 ± 0.0	0.0 ± 0.0	9.5^{a} ± 0.6	38.9^{b} ± 1.6	0.0 ± 0.0	63^{c} ± 2	0.0 ± 0.0	0.0 ± 0.0
E. coli	4.36nd ± 0.04	35^{b} ± 2	5.69^{a}± 0.13	56^{c} ± 2	0.0 ± 0.0	51.8^{bc} ± 0.8	0.0 ± 0.0	0.0 ± 0.0
K. pneumoniae	5.2^{a} ± 0.9	13.7^{a} ± 0.8	0.00 ± 0.00	18.5^{a} ± 1.0	0.0 ± 0.0	46.3^{b} ± 1.0	0.0 ± 0.0	37.9^{b} ± 1.2
L. monocytogenes	27.2^{b} ± 1.1	42^{b} ± 2	32.6^{b} ± 1.2	33.1^{b} ± 1.3	38.5^{e} ± 1.2	50^{b} ± 1	0.0^{a} ± 0.0	0.0 ±0.0
P. aeruginosa	0.0 ± 0.0	20.0^{a} ± 1.0	5.8^{a} ± 0.2	51.5^{b} ± 1.7	0.0^{a} ± 0.0	47.7^{b} ± 1.1	0.0 ± 0.0	0.0 ± 0.0
S. aureus	49.8^{b}± 1.7	65^{c} ± 3	48^{b}± 2	49.4^{b} ± 1.9	50.1^{b} ± 1.2	59.7^{c} ± 0.6	0.0 ± 0.0	0.0 ± 0.0
**MTT24**	**L-SM**		**L-UAE**		**S-60**		**S-40**	
	**0.5 mg/mL**	**1 mg/mL**	**0.5 mg/mL**	**1 mg/mL**	**0.5 mg/mL**	**1 mg/mL**	**0.5 mg/mL**	**1 mg/mL**
A. baumannii	0.0 ± 0.0	0.0 ± 0.0	0.0 ± 0.0	0.0 ± 0.0	0.0 ± 0.0	60^{c} ± 2	0.0 ± 0.0	0.0 ± 0.0
E. coli	0.0 ± 0.0	7.5^{a} ± 0.6	0.0 ± 0.0	0.0 ± 0.0	0.0 ± 0.0	48.3^{b} ± 1.7	0.0 ± 0.0	0.0 ± 0.0
K. pneumoniae	0.0 ± 0.0	0.0 ± 0.0	0.0 ± 0.0	0.0 ± 0.0	0.0 ± 0.0	41^{b} ± 2	0.0 ± 0.0	30.3^{b} ± 1.9
L. monocytogenes	0.0 ± 0.0	0.0 ± 0.0	0.0 ± 0.0	1.29nd ± 0.08	0.0 ± 0.0	37^{b} ± 2	0.0 ± 0.0	0.0 ± 0.0
P. aeruginosa	0.0 ± 0.0	0.0 ± 0.0	0.0 ± 0.0	0.0 ± 0.0	19.6^{a}± 1.3	52^{c} ± 3	0.0 ± 0.0	0.0 ± 0.0
S. aureus	0.0 ± 0.0	17.8^{a} ± 0.7	45.4^{b} ± 1.1	64.0^{c} ± 1.3	45^{b} ± 2	56^{c} ± 3	0.0 ± 0.0	0.0 ± 0.0

Results are presented as percentages, assuming for the control (untreated bacteria) an inhibition = 0. Sample concentration: 0.5 and 1 mg/mL. Samples: leaf-derived extract macerated for 5 minutes with EtOH 40% (L-SM); leaf-derived extract obtained by ultrasound treatment for 5’ with EtOH 40% (L-UAE), stems-derived extract obtained by ultrasound treatment for 5” with EtOH 60% (S-60) and stems-derived extract obtained by ultrasound treatment for 5” with EtOH 40% (S-40). Note: equal letters denote no significant difference; different letters denote significant differences (one-way ANOVA + Tukey HSD, $p < 0.05$; n = 3).
